# CDK6 as a Biomarker for Immunotherapy, Drug Sensitivity, and Prognosis in Bladder Cancer: Bioinformatics and Immunohistochemical Analysis

**DOI:** 10.7150/ijms.101043

**Published:** 2024-09-16

**Authors:** Xiaojie Zhao, Xin Yu, Wenge Li, Zhiyuan Chen, Tingting Niu, Xiaodong Weng, Lei Wang, Xiuheng Liu

**Affiliations:** 1 Department of Urology, Renmin Hospital of Wuhan University, 430060 Wuhan, Hubei, China.; 2 Department of Breast and Thyroid Surgery, Renmin Hospital of Wuhan University, 430060 Wuhan, Hubei, China.; 3 Department of Oncology, Shanghai Artemed Hospital, 200131 Shanghai, China.; 4 Tongji University Cancer Center, Shanghai Tenth People's Hospital, School of Medicine, Tongji University, Shanghai 200072, China.

**Keywords:** cyclin-dependent kinase (CDK6), bladder cancer, immunotherapy, drug sensitivity, prognostic prediction

## Abstract

**Background:** CDK6 is linked to tumor progression and metastasis, although its molecular mechanism and prognostic value are unclear in bladder cancer.

**Materials and methods:** In our study, raw data were obtained from public databases and Single-center retrospective case series. We conducted a bioinformatics analysis and immunohistochemistry to explore the biological landscape of CDK6 in tumors, with a particular focus on bladder cancer. We examined its expression characteristics and prognostic value and performed functional annotation analysis using gene function enrichment. We also assessed the association between bladder cancer molecular subtypes and mutation spectra and analyzed the landscape of the tumor immune microenvironment to predict treatment response sensitivity.

**Results:** Our study found that CDK6 was a potential prognostic marker for bladder cancer. We discovered that bladder cancer patients with high CDK6 expression do not respond well to immunotherapy and have a poor prognosis. CDK6 regulates tumor immune status, metabolism, and cell cycle-related signaling pathways, thereby influencing tumor biological behavior. Furthermore, CDK6 mediated the suppression of the immune microenvironment to weaken anti-tumor immune responses. Finally, a comprehensive characterization of CDK6 was applied in the prognostic prediction of bladder cancer, suggesting that targeting CDK6 represents a potential therapeutic option.

**Conclusions:** These results indicated that CDK6 is an independent biomarker for predicting prognosis and immunotherapy efficacy of bladder cancer. A deeper understanding of its specific molecular mechanisms may provide new treatment strategies.

## 1. Introduction

In the urinary system, bladder cancer (BLCA) remains the second most common type of tumor with increasing trend in incidence worldwide^1^. It was estimated that in 2024 there will be approximately 83190 cases of bladder cancer and approximately 16840 deaths related to bladder cancer in the United States^2^. Benefiting from advancements in disease management such as surgical treatment, adjuvant systemic therapy, immunotherapy, and targeted therapy, the 5-year survival rates of bladder cancer have improved^3^. Nevertheless, Immunotherapeutic strategies are beneficial to a small percentage of patients, but most patients experience immunotherapy resistance^4^. Discovering new tumor immunotherapy targets and effective biomarkers through a deep understanding of the molecular mechanisms underlying bladder cancer is crucial for its diagnosis and treatment.

The family of cyclin-dependent kinases (CDKs) contains multiple members. When bound to their respective matched cyclins, CDKs are subsequently catalytically activated to regulate cell-cycle progression and transcription^5^. CDK6, known as classic cell cycle kinases, participates in cell cycle regulation and transcriptional regulation by forming complexes with D-type cyclins^6^, ^7^. In recent years, CDK6 has gradually been recognized as a core regulator in cellular signaling pathways leading to cancer development and as a major driver of cancer progression. Studies show that various tumors are associated with CDK6, including hematological malignancies, breast cancer, and melanoma 8. In addition, CDK6 has been implicated in the regulation of immune cell infiltration and immune checkpoint molecule expression, thereby influencing the immune response against tumors^9^. For instance, CDK6 inhibition has shown potential in enhancing the efficacy of immune checkpoint inhibitors by altering the tumor microenvironment to be more conducive to immune cell infiltration and activity^10^, ^11^. Given the important function of CDK6 in virous tumors, the exploration of potential therapeutic targets and novel biomarkers brings hope not only for prognosis prediction but also for therapeutic interventions.

This study aims to identify the predictive value of CDK6 in bladder cancer by analyzing its phenotypic characteristics, assessing its immune-system impact, and developing a prediction model based on these findings. We hope to provide a new insight for therapies of bladder cancer.

## Materials and methods

### 2.1 Patients and specimens

Cohort1: A gene symbol was generated from the gene expression data for 408 TCGA-BLCA samples, along with information on gene mutations and clinicopathology^12^. As an expression level, the average value is calculated when more than one probe matched a gene symbol. Table S1 provides a description of the characteristics of patients in cohort 1.

Cohort2: In the period between February 2018 and October 2022, 100 formalin-fixed paraffin-embedded specimens of bladder cancer patients were collected from the Renmin Hospital of Wuhan University. An institutional ethics committee approval was obtained for this study from Renmin Hospital of Wuhan University (WDRY2022-K077). As defined by the eighth edition of the AJCC bladder cancer staging system, clinical features include age, sex, and clinical stage.

Cohort3: BLCA immunotherapy-related data, including expression data and clinical information from 348 patients, were obtained by using the R package IMvigor210 CoreBiologies for BLCA immunotherapy-related data^13^. The characteristics of patients in cohort 3 are provided in Table S3.

Cut-off Value Determination: For both the TCGA (Cohort 1) and IMvigor (Cohort 3) cohorts, bulk RNA-seq data were used. The cut-off values for gene expression were determined using the survminer R package, which identified the optimal threshold for stratifying patients based on gene expression levels. In both cohorts, the cut-off value was established at 13.77277.

### 2.2 Survival studies

Based on Kaplan-Meier analysis, survival R packages were used for survival studies. We employed univariate Cox regression analysis to determine whether age, molecular subtype, and stage, as well as CDK6 expression, are associated with the prognosis of patients with BLCA. An expanded Cox regression model using Survival R was used to assess the significant prognostic factors identified in univariate Cox regression analysis at p <0.05.

### 2.3 Differential expressed genes screening and functional exploration

Using the Limma R package, downregulated and upregulated DEGs were screened under P <0.05 and fold change > 1.5 conditions. For enrichment analysis, the ClusterProfiler package was used to assess Gene Ontology (GO) and KEGG pathway information. To account for multiple comparisons and ensure the robustness of the results, we applied the Benjamini-Hochberg method to calculate the false discovery rate (FDR) for P-value correction in both differential gene expression and pathway enrichment analyses.

### 2.4 Annotation for molecular subtype of bladder cancer

Based on ConsensusMIBC R packages, we analyzed bladder cancer molecular subtypes in a range of molecular subtype systems, including TCGA, Baylor, CIT, Lund, MDA, and UNC.

### 2.5 Immune cell infiltration analysis

With the help of the CIBERSORT R package, 22 immune cells were calculated as part of the infiltration of tumors, via the CIBERSORT algorithm.

### 2.6 Chemotherapy reaction forecast

IC50s of commonly used chemotherapeutic drugs were measured using R's "pRRophetic" package, while drug target genes were screened from the Drugbank database (https://go.drugbank.com/).

### 2.7 Immunohistochemical analysis

As detailed in our previous study, IHC staining was performed by two independent pathologists. Based on the percentage and intensity of positively stained tumor cells, CDK6 expression scores were calculated as follows: 0 (negative), 1 (weak positive, light brown), 2 (moderate positive, brown), and 3 (strong positive, dark brown). In order to calculate the protein staining score, we used the formula: staining score = percentage score × intensity score. The determination of the cut-off value for the CDK6 protein staining score was based on the median value obtained from immunohistochemistry (IHC) analysis. Specifically, the median score, derived from the distribution of staining intensities across the entire cohort, was used to dichotomize patients into high and low CDK6 expression groups. In this study, the calculated cut-off value was 27.931, which serves as a critical threshold for stratifying patients according to their CDK6 expression levels. The antibody used in this assay was anti-CDK6 (Cat No. 14052-1-AP, Proteintech, China).

### 2.8 Statistical analysis

When examining the correlations between variables, Pearson correlation analysis played a vital role. We used a t-test to compare continuous variables between binary groups following a normal distribution, and Kruskal-Wallis tests to compare differences among more than two groups. Statistically significant differences were identified by the log-rank test, and survival curves for subgroups were generated by the Kaplan-Meier method. It was calculated two-sided using SPSS soft v22.0, SangerBox website (www.sangerbox.com), and R studio 4.0.0, and statistical significance was determined if the P value was less than 0.05.

## 3. Results

### 3.1 The prognostic prediction value and mutation characteristic of CDK6 in pan-cancer

**Figure 1:**
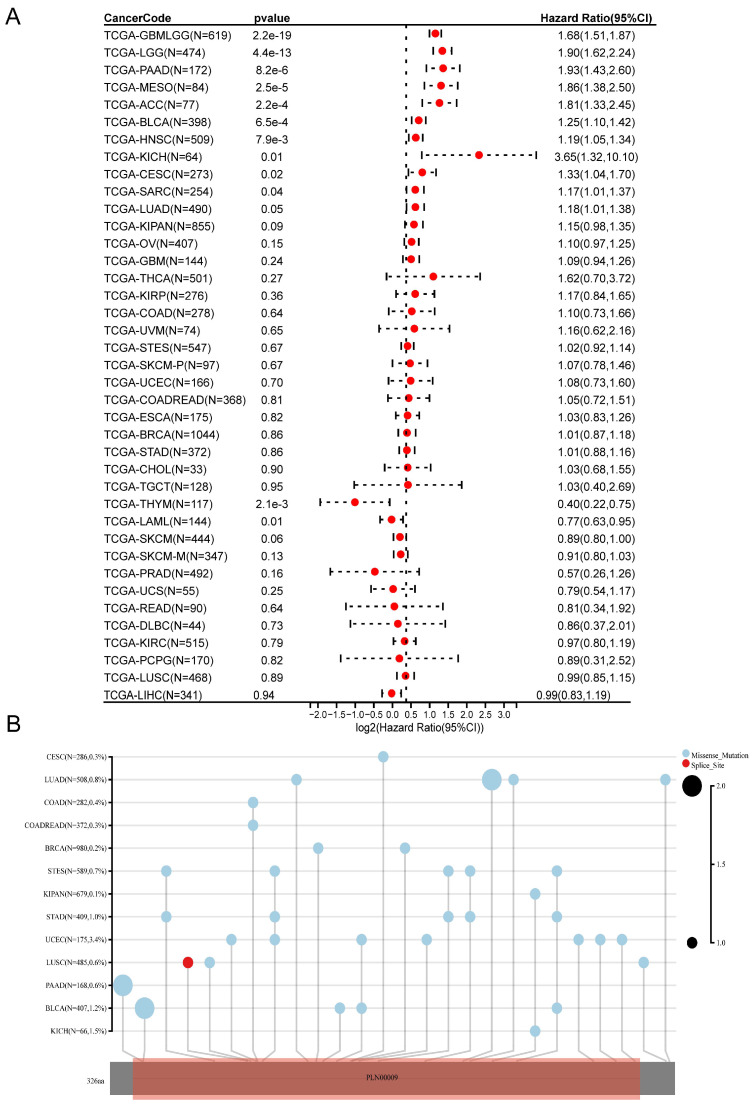
** Correlations of CDK6 with prognosis and mutation in pan-cancer.** (A) Forest plot of associations between CDK6 expression and overall survival in pan-cancer. (B) Map position of the CDK6 mutation in pan-cancer.

An evaluation of CDK6 expression and the clinical outcome was carried out using Kaplan-Meier survival analysis to assess CDK6's prognostic assessment value in pan-cancer. To begin with, we investigated the relationship between CDK6 expression and OS in 39 cancers, finding that aberrant CDK6 expression was associated with OS in GBML, LGG, PAAD, MESO, ACC, and BLCA, where high expression of CDK6 was correlated with worse prognosis (Figure 1A). The mutation sites and types of CDK6 in pan-cancer were also analyzed. It was notable that UCEC had the most mutation sites, while BLCA had a significant level of enrichment. (Figure 1B).

### 3.2 Prognostic value of the CDK6 expression in bladder cancer

**Figure 2:**
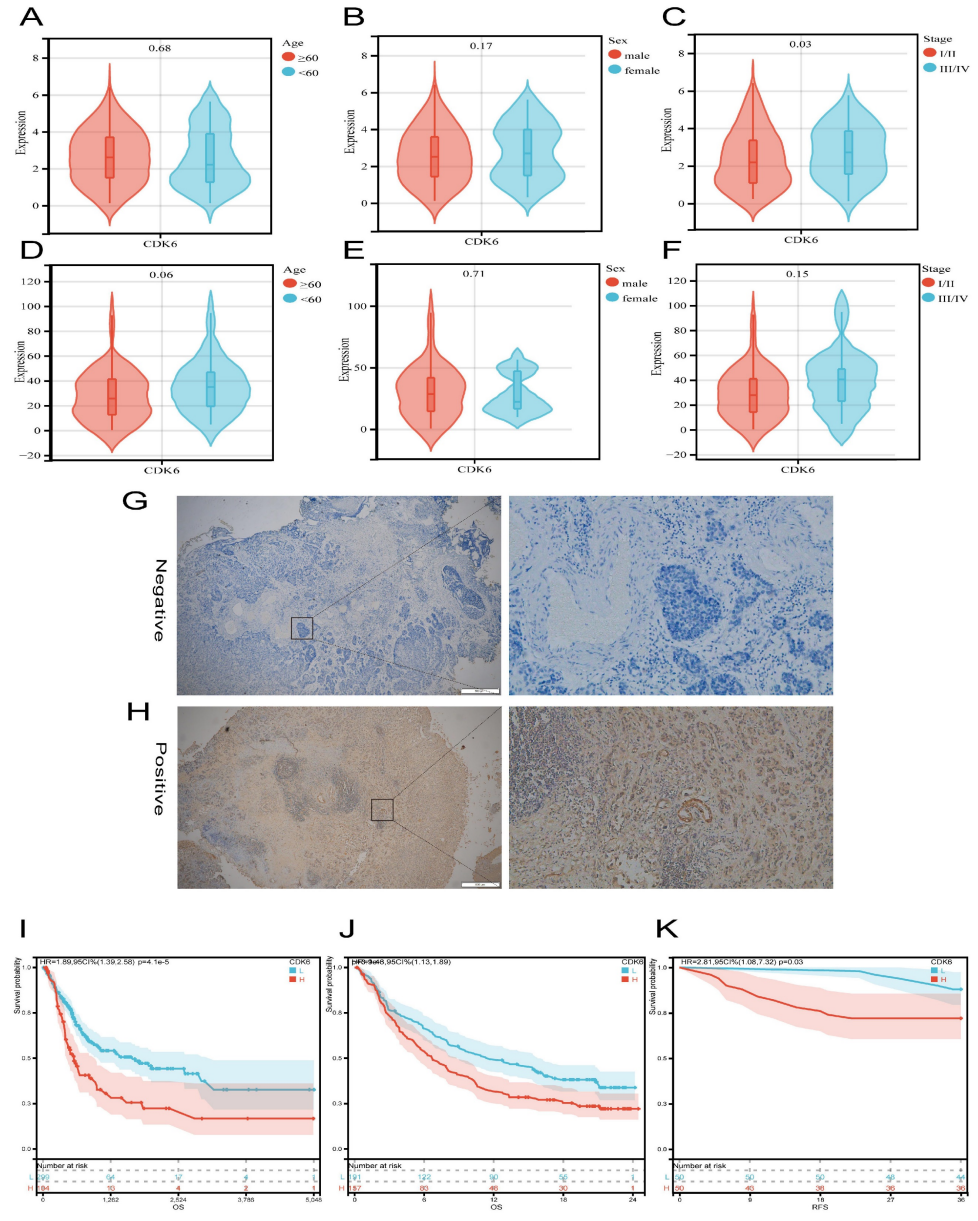
** Analysis of the prognostic value of CDK6 in bladder cancer.** (A-C) CDK6 expression in different (A) Age, (B) Sex and (C) Stage in cohort1. (D-F) CDK6 expression in different (D) Age, (E) Sex and (F) Stage in cohort2. (G, H) Representative immunohistochemistry images for CDK6 in bladder cancer. (I-K) OS for CDK6 in (I) cohort1 and (J) cohort3, RFS for CDK6 in (K) cohort2.

Firstly, we analyzed the expression and clinical features of CDK6 in patients in cohort 1. According to the results, patients with early pathological stages had low CDK6 expression, whereas late-stage patients had high CDK6 expression. Despite this, there were no significant differences in CDK6 expression between patients of different genders and ages in cohort 1(Figure 2A-C). Similarly, data from cohort 2 showed that there were no obvious differences (Figure 2E-F). Additionally, CDK6 expression was examined in 100 patients with BLCA by IHC in order to confirm that this expression is associated with a better prognosis in cohort 2, while in cohort 1, the expression was not (Figure 2G-H). The result of IHC showed that CDK6 localized diffusely in the cytoplasm of BLCA cells. Cohort 1 Kaplan-Meier analysis revealed a negative association between CDK6 expression and OS (Figure 2I). By analyzing data from cohort 3, we validated the correlation between CDK6 expression and immunotherapy response (Figure 2J). Similarly, we validated the relationship between CDK6 expression and progression-free survival (PFS) in Cohort 2(Figure 2K). As a result of these findings, CDK6 might be an indicator of poor prognosis for BLCA patients.

### 3.3 Discovery of DEGs and functional annotations

**Figure 3:**
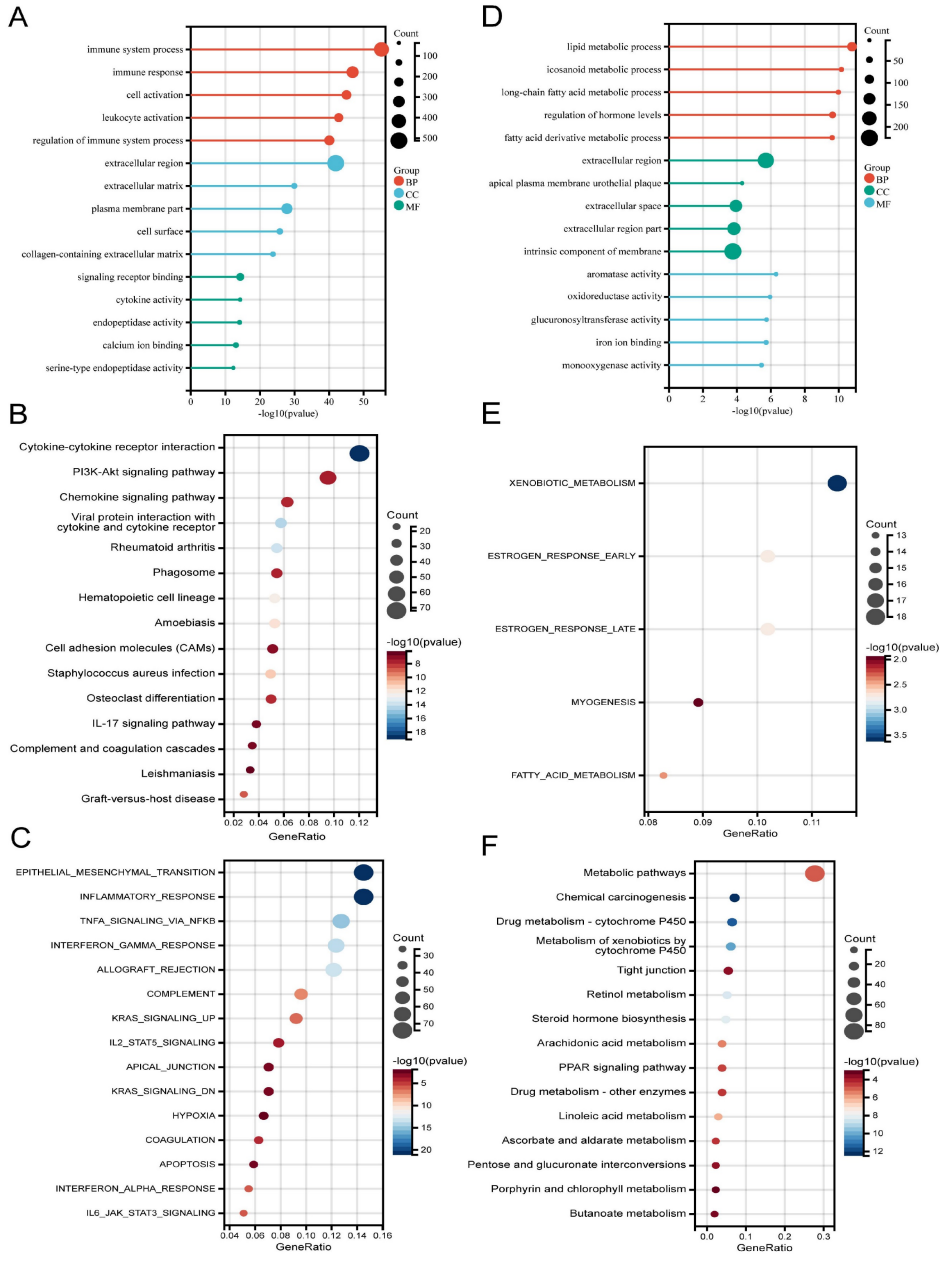
** Functional analysis for CDK6 in bladder cancer.** (A-C) (A) GO, (B) KEGG and (C) Hallmark pathways of upregulated DEGs. (D-F) (D) GO, (E) KEGG and (F) Hallmark pathways of downregulated DEGs.

According to the DEG analysis, patients with high CDK6 expression had 2093 upregulated DEGs and 1306 downregulated DEGs compared to patients with low CDK6 expression (Figure S1A-B). Afterward, functional annotations of DEGs were performed. According to GO analysis, upregulated DEGs disproportionately enriched in "Immune system process" (in BP), "Extracellular region" (in CC), and "Signaling receptor binding" (in MF) (Figure 3A). KEGG analysis revealed that upregulated DEGs were mainly involved in "Cytokine-cytokine receptor interaction" and "PI3K-Akt signaling pathway" (Figure 3B). Hallmark gene set analysis revealed that "Epithelial-mesenchymal transition" and "Inflammatory response" were highly enriched (Figure 3C).

For the downregulated DEGs, GO analysis manifested that the most substantially enriched pathways were Lipid metabolic process" in BP, “Extracellular region” in CC, and "Aromatase activity" in MF (Figure 3D). For the KEGG pathways, significantly enriched pathways were "Metabolic pathways", "Chemical carcinogenesis" and "Xenobiotics metabolism by cytochrome P450", etc. (Figure 3E). In the hallmark gene set analysis, genes related to "Xenobiotic metabolism", "Estrogen response early", and "Estrogen response late" were found to be enriched (Figure 3F).

Based on these results, CDK6 appears to be associated with bladder cancer differentiation characteristics and immunity.

### 3.4 Correlation of CDK6 expression with molecular typing of bladder cancer

**Figure 4:**
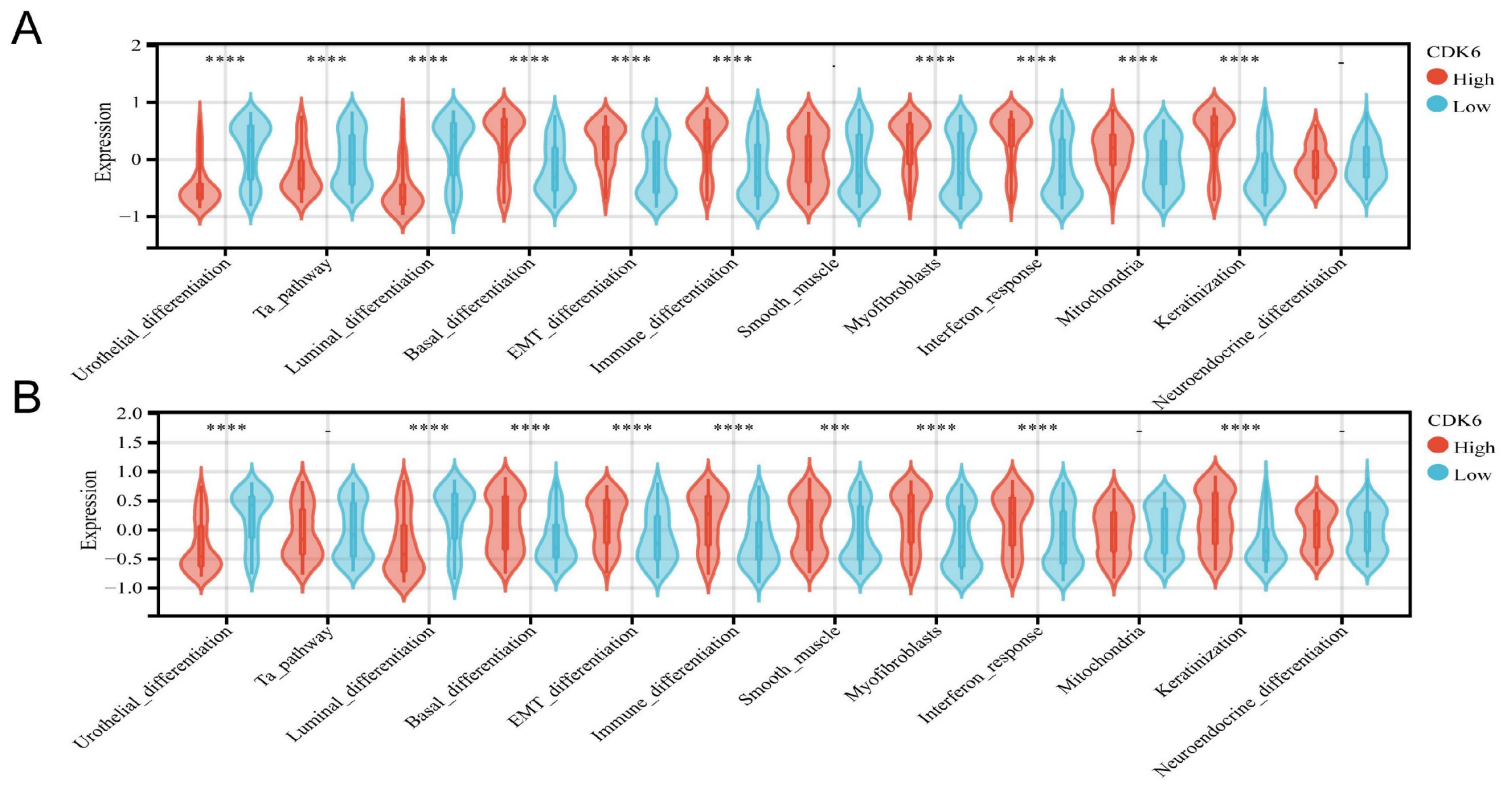
** Correlation analysis between CDK6 and molecular subtype in bladder cancer.** (A, B) Differential expression of specific bladder cancer-related signatures in high and low CDK6 expression group patients in (A) cohort 1 and (B) cohort3. ***P < 0.001 and ****P < 0.0001.

It has been shown that the prognosis and treatment response of bladder cancer vary depending on the molecular subtype. Cohort 1 examined the relationship between CDK6 and molecular subtypes of bladder cancer (table S4), and a comprehensive analysis revealed that bladder cancer with high expression of CDK6 is more likely to be of the basal type, using different molecular subtype prediction algorithms. It was confirmed by molecular characteristics that patients with high CDK6 had higher basal differentiation, EMT differentiation, immune differentiation, myofibroblasts, interaction response, mitochondria, and keratinization but lower urothelial differentiation, Ta pathway and Luminal differentiation (Figure 4A).

The same analyses were applied in the cohort 3 (Table S5). And the result showed that high CDK6 expression group exhibited higher levels of basal, EMT, and immune differentiation, smooth muscle differentiation, myofibroblast presence, interaction response, and keratinization, but showed lower levels of urothelial differentiation and Luminal differentiation (Figure 4B). There was a significant association of high CDK6 with infiltrating molecular type which may lead to a worse prognosis.

### 3.5 Association between CDK6 and tumor immune microenvironment

**Figure 5:**
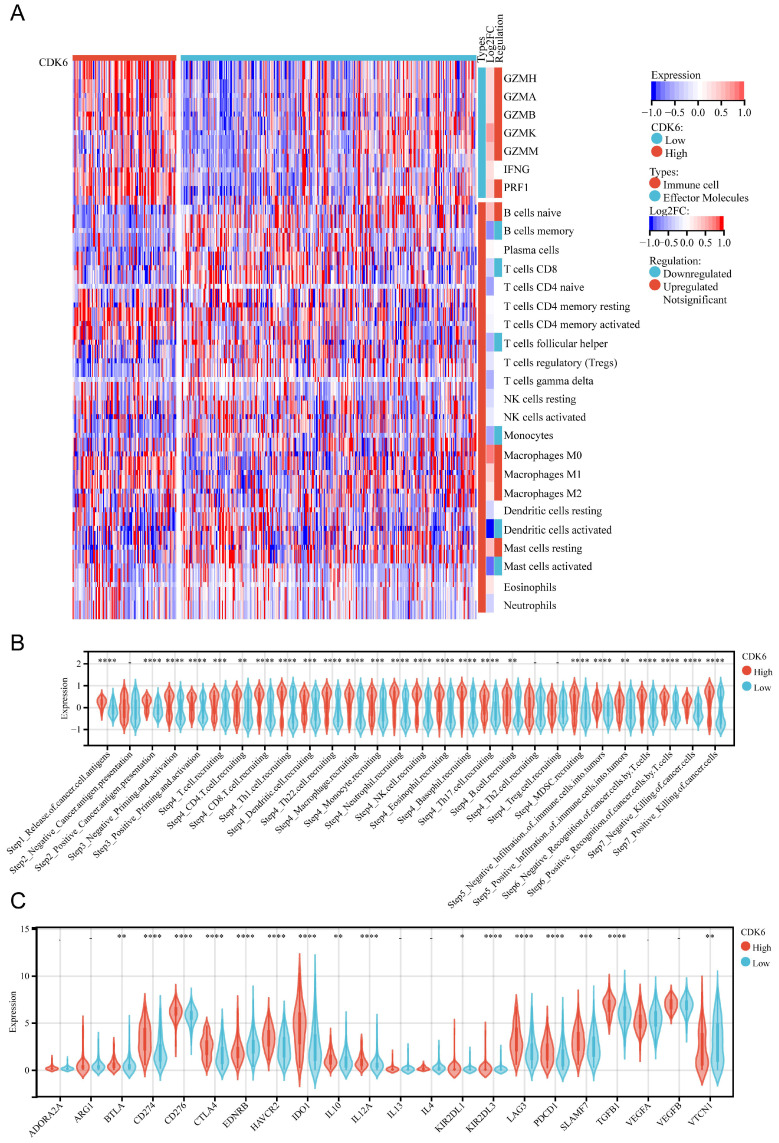
** Correlation analysis between CDK6 and tumor immune microenvironment in cohort 1.** (A) Heatmap of immune cells in high and low CDK6 expression group (B-C) Correlation violin plot of (B) immune cycle and (C) immune checkpoints genes in high and low CDK6 expression group. *P < 0.05, **P < 0.01, ***P < 0.001 and ****P < 0.0001.

In cohort 1, we investigated the relationship between CDK6 and immunological features, as well as the cycle of cancer immunity. To deconvolute the differences in the types and levels of immune cell infiltration within the tumor immune microenvironment (TIME), the CIBERSORT algorithm was applied. For effector molecules, patients with high expression of CDK6 expressed higher levels of GZMH, GZMA, GZMB, GZMK, GZMM, and PRF1 compared to patients with low expression of CDK6, although IFNG levels were not significantly changed. Regarding immune cell infiltration, it was observed that high CDK6 expression group had less infiltration of memory B cells, CD8+ T cells, follicular helper T cells, monocytes, activated dendritic cells, and activated mast cells, and more infiltration of M0 macrophages, M1 macrophages, M2 macrophages, and resting mast cells (Figure 5A). The high-CDK6 group could activate a majority of steps in the immunity cycle, except for cancer antigen presentation (Step 2) and trafficking of immune cells to tumors (Step 4), including Th2 cell recruiting and Treg cell recruiting (Figure 5B). Furthermore, the violin plots showed that the vast majority of the immune-relevant pathways were significantly activated in correlation with CDK6 expression (Figure 5C).

**Figure 6:**
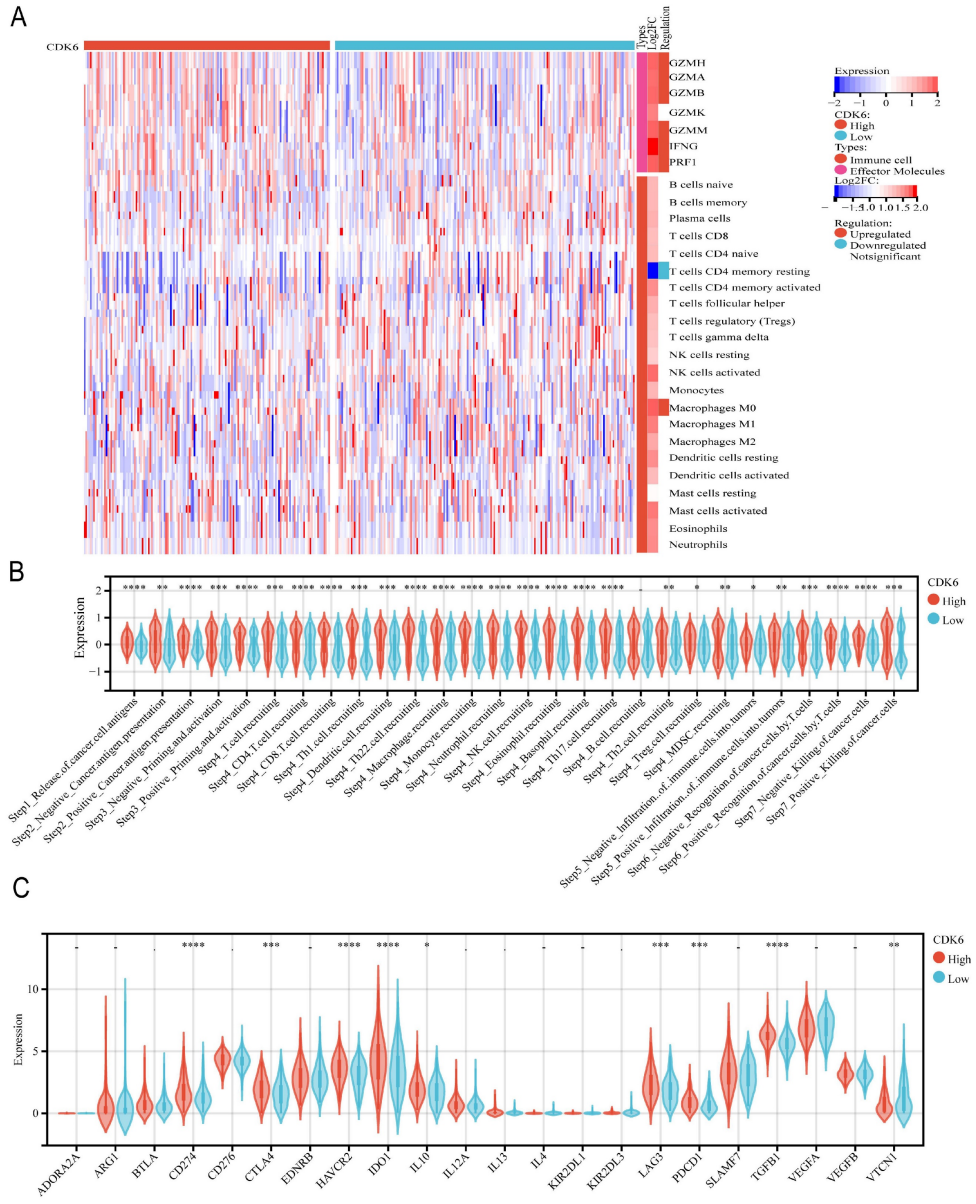
** Correlation analysis between CDK6 and tumor immune microenvironment in cohort 3.** Heatmap of immune cells in high and low CDK6 expression group (B-C) Correlation violin plot of (B) immune cycle and (C) immune checkpoints genes in high and low CDK6 expression group. *P < 0.05, **P < 0.01, ***P < 0.001 and ****P < 0.0001.

The same analysis was performed for cohort 3. For effector molecules, we observed that the expression of molecules such as GZMH, GZMA, GZMB, GZMM, IFNG, and PRF1 was significantly enriched, except for GZMK. Regarding immune cell infiltration, we observed less infiltration of resting memory CD4+ T cells and more infiltration of M0 macrophages (Figure 6A). All steps in the immunity cycle, except B cell recruiting, were activated (Figure 6B). Moreover, several pathways significantly correlated with tumor progression were activated in the high CDK6 expression group, including the IFNG signature, APM signal, Fanconi anemia pathway, homologous recombination, p53 signaling pathway, and spliceosome, which are recognized to be involved in tumor initiation, progression, and immunotherapy resistance (Figure 6C).

### 3.6 Immunological correlation of CDK6 and pan-cancer

Since CDK6 was found to exhibit the most obvious immunosuppression in the tumor immune microenvironment (TIME) in BLCA, our next analysis focused on the influence of CDK6 expression in pan-cancer. First, we assessed the relationship between CDK6 levels and immune regulatory genes, including chemokines, receptors, MHC, immunoinhibitory, and immunostimulatory genes. The results showed that CDK6 is closely correlated with regulatory genes in multiple cancers. Although there were varied correlations among different cancers, CDK6 exhibited the most significant positive correlation with BLCA (Figure S2A). After analyzing the relationship between CDK6 and immune checkpoint genes in pan-cancer, we found significant correlations between CDK6 expression and 60 immune checkpoint genes. There was a significant positive correlation between CDK6 expression and immune checkpoint gene expression in BLCA (Figure S2B). To further identify the relationship between CDK6 expression and the immune microenvironment in pan-cancer, we investigated the heatmap of correlation between CDK6 expression and the infiltration of 22 different types of immune cell subtypes. Different infiltrating immune cells were found to be significantly heterogeneous in pan-cancer, especially in BLCA (Figure S2C).

### 3.7 CDK6 predicts treatment response

**Figure 7:**
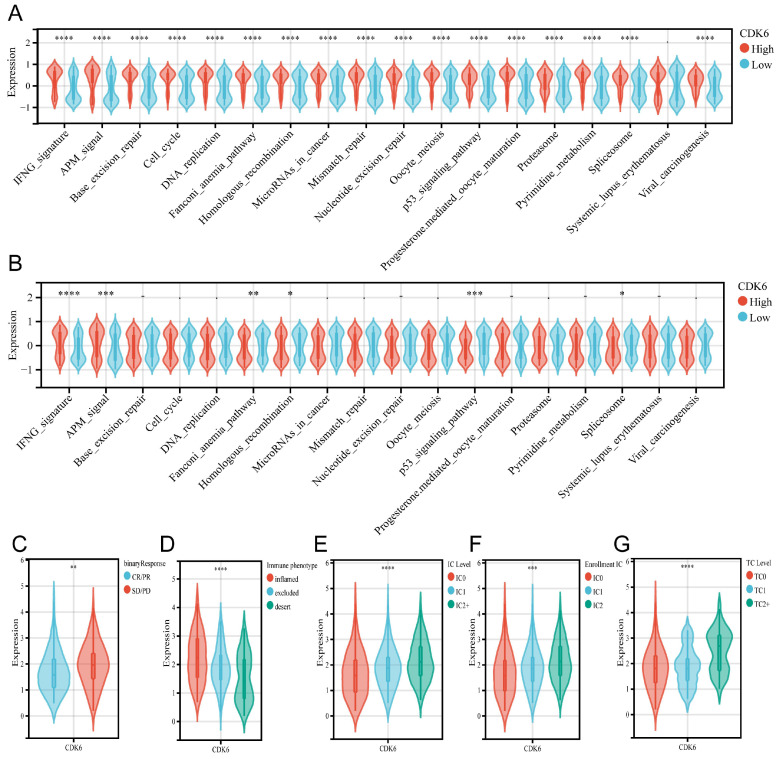
** Predicting immunotherapy response with CDK6.** (A.B) Identifying how CDK6 regulates immune response-related pathways in cohorts 1 (A) and 3 (B). (C-F) Detection of CDK6 expression in patients with different clinical responses to (C) tumor immunotherapy, (D) types of immune cell infiltration, (E) IC levels, (F) enrollment IC, and (G) tumor cell counts. *P < 0.05, **P < 0.01, ***P < 0.001 and ****P < 0.0001.

To evaluate the predictive role of CDK6 in immunotherapy for bladder cancer, we assessed its association with various immune-related pathways. In cohort1, CDK6 showed a positive correlation with all immune predictive pathways, except for the Systemic Lupus Erythematosus pathway (Figure 7A). However, in cohort3, the results were markedly different. CDK6 was positively correlated only with the IFNG and APM pathways, while it was negatively correlated with the Fanconi Anemia Pathway, Homologous Recombination, p53 Signaling Pathway, and Spliceosome. CDK6 showed no significant correlation with other immune predictive pathways in cohort3(Figure 7B). Furthermore, data from cohort 3 showed that patients who received immunotherapy with poor results expressed higher levels of CDK6 (Figure 7C). Despite higher immune cell infiltration and increased immune checkpoint expression in high CDK6 expressing tumors, the overall immune response is suppressed, leading to poorer outcomes in immunotherapy (Figure 7D-G).

### 3.8 Molecular mechanisms underlying CDK6 and radiotherapy, chemotherapy, and targeted therapy for bladder cancer

**Figure 8:**
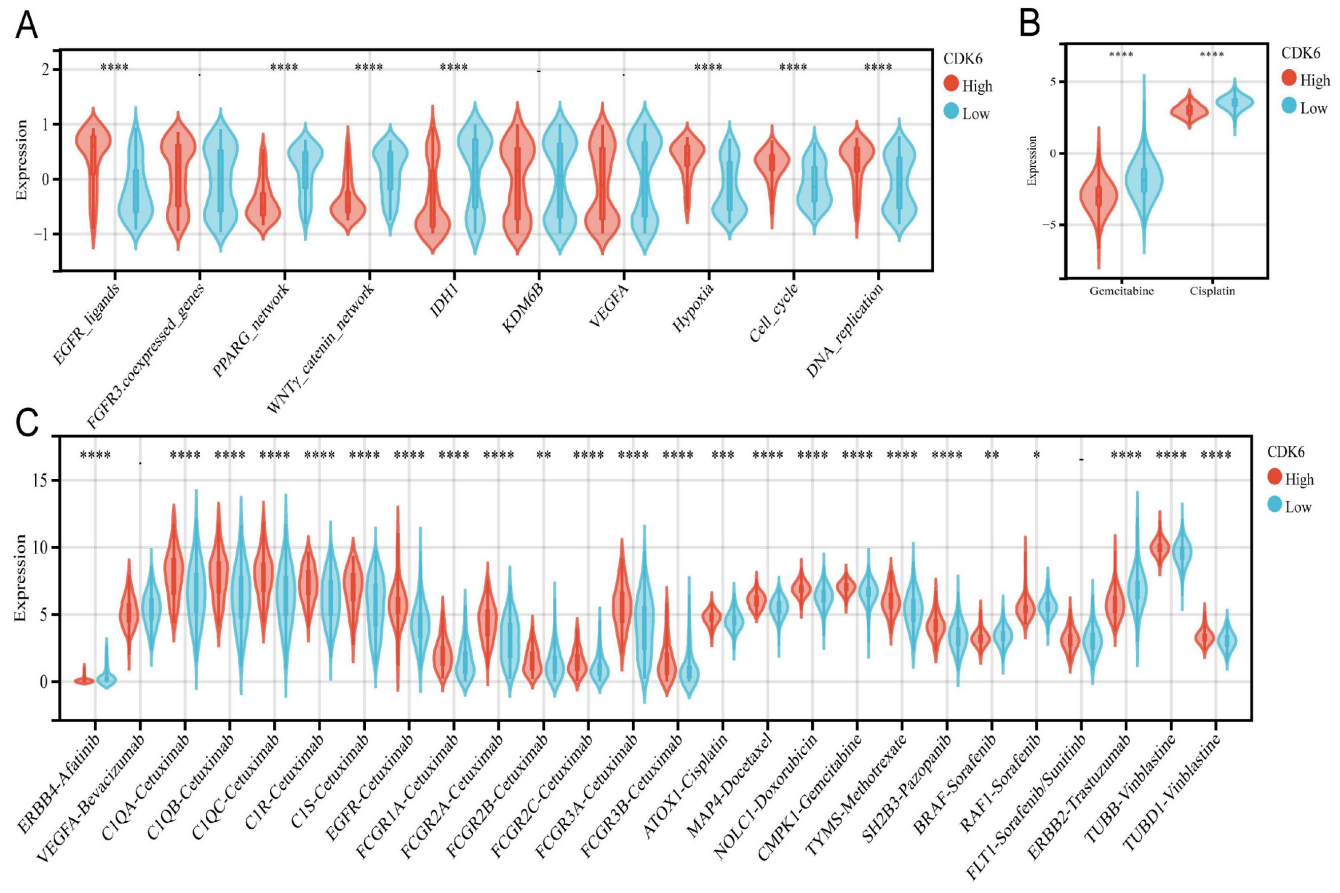
** Correlation between CDK6 and therapeutic response in cohort 1.** (A-C) Differential expression of enrichment scores of therapeutic signatures such as (A) targeted therapy and radiotherapy, (B) IC50 of gemcitabine and Cisplatin therapy, (C) drug-target genes in high and low CDK6 expression group. *P < 0.05, **P < 0.01, ***P < 0.001 and ****P < 0.0001.

**Figure 9:**
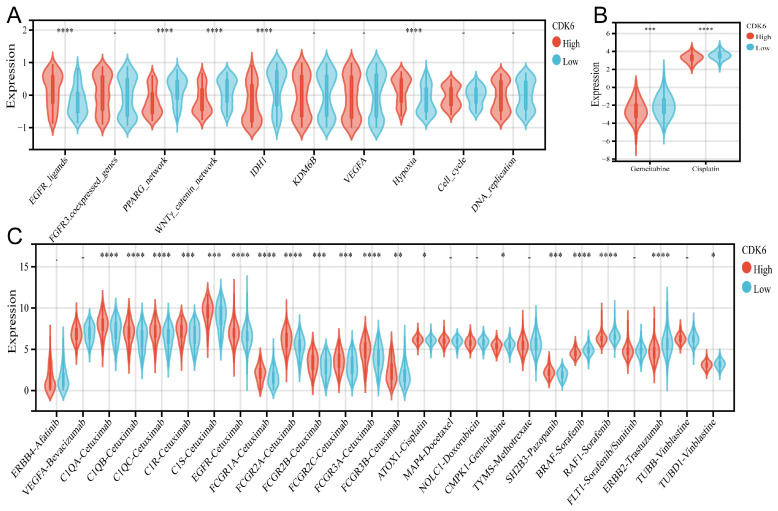
** Correlation between CDK6 and therapeutic response in cohort 3.** (A-C) Differential expression of enrichment scores of therapeutic signatures such as (A) targeted therapy and radiotherapy, (B) IC50 of gemcitabine and Cisplatin therapy, (C) drug-target genes in high and low CDK6 expression group. *P < 0.05, **P < 0.01, ***P < 0.001 and ****P < 0.0001.

A comparison of the clinical responses to chemotherapy, radiotherapy, and numerous targeted therapies was conducted next. There were differences and similarities in the treatment responses between cohorts 1 and 3. The high CDK6 expression group in TCGA showed higher enrichment scores for the EGFR network (EGFR ligands) and radiotherapy-predicted pathways (hypoxia, cell cycle, and DNA replication), while the low CDK6 expression group expressed higher enrichment for pathways inhibiting oncogene production (PPARG pathway, WNT/β-catenin pathway and IDH1 pathway) (Figure 8A). The group in cohort 3 showed similar results (Figure 9A). The pRRophetic calculation revealed that the low CDK6 expression group in cohort 1 and cohort 3 were more sensitive to gemcitabine and cisplatin, the two most common chemotherapeutic agents used to treat bladder cancer (Figure 8B, Figure 9B). Subsequently, Drugbank database results showed that the high CDK6 expression group in cohort 1 presented higher levels of molecular targeted agents, including tyrosine kinase inhibitors, BRAF inhibitors, biologics encompassing monoclonal antibodies, and enzyme modulators (Figure 8C), which showed more extensive activation than cohort 3 (Figure 9C). These findings highlight the potential of CDK6 as a biomarker for predicting treatment responses in bladder cancer, suggesting that targeting CDK6 could optimize the efficacy of chemotherapy, radiotherapy, and targeted therapies.

### 3.9 CDK6 and Mutational Landscape

**Figure 10:**
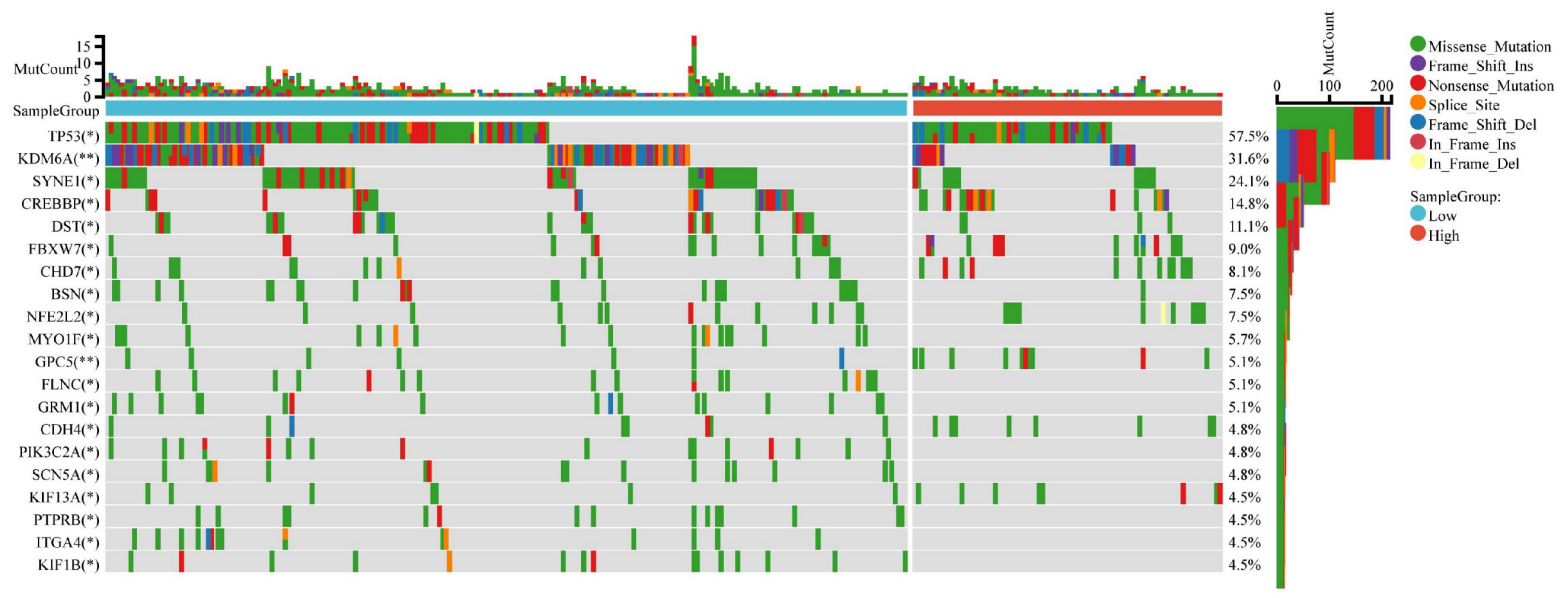
** Mutational landscape for CDK6 high group and CDK6 low group.** Comparison of the mutational landscape between CDK6 high groups and CDK6 low groups. *P < 0.05 and **P < 0.01.

The mutational landscape of bladder cancer samples was compared between the CDK6 high expression group and the CDK6 low expression group. The overall mutation count (MutCount) for each sample is depicted in the top bar plot, with different colors representing various mutation types. The heatmap illustrates the distribution of genetic mutations in several key genes across the two groups. TP53 exhibits the highest mutation frequency, with a mutation rate of 57.5%. Other frequently mutated genes include KDM6A (31.6%), SYNE1 (24.1%), CREBBP (14.8%), and DST (11.1%) (Figure 10).

These findings highlight the distinct mutational landscapes in bladder cancer based on CDK6 expression levels. The significant association between CDK6 expression and specific genetic alterations suggests that CDK6 may serve as a valuable biomarker for stratifying patients and guiding targeted therapeutic strategies.

## 4. Discussion

Bladder cancers exhibit significant heterogeneity with variable molecular subtypes, each possessing distinct clinical and biological characteristics^14^. This heterogeneity necessitates the identification of new molecular biomarkers for personalized treatment strategies in bladder cancer management^15^. There is a growing body of evidence to suggest that selective inhibition of CDK6 function as a transcriptional regulator could become a novel therapeutic approach^8^. Several previous studies have shown that the CDK6, a downstream target gene of multiple miRNAs, is down-regulated to promote tumorigenesis and development of bladder cancers through multiple mechanisms^16^-^18^. A previous study found that circLAMA3 binds to MYCN mRNA to affect DNA replication by downregulating CDK6, Inhibiting bladder cancer proliferation by arresting the cell cycle at G0/G1^19^. Taken together, CDK6 inhibitors have demonstrated promising anticancer potential. However, previous studies are often limited to specific gene functions of CDK6, lacking comprehensive assessment based on functional and molecular status in tumors.

Benefiting from rapid development of bioinformatics and sequencing technologies, extensive findings and analyses of novel cancer biomarkers have expanded the perspective of personalized cancer therapy, providing us with guidance for precision therapy^20^, ^21^. Tumor biomarkers play a crucial role in guiding treatment decisions and predicting responses in various cancers. For example, established biomarkers based on pathway-level and tumor mutational burden have significantly improved patient management and treatment outcomes^22^, ^23^. Given this context, CDK6 emerges as a promising biomarker in bladder cancer, with the potential to similarly inform therapeutic strategies and enhance personalized treatment approaches. Our study aims to confirm that CDK6 is a critical molecule involved in multiple cancer-promoting mechanisms related to bladder cancer prognosis.

Bladder cancer is often associated with distinct epigenetic and immunological alterations, including aberrant DNA methylation patterns. Studies have shown that these methylation changes can influence tumor behavior and contribute to disease progression, providing valuable insights into diagnostic and therapeutic strategies for bladder cancer^24^, ^25^. Since different molecular subtypes exhibit significant heterogeneity, it is important to make prognoses and responses to therapy more specific and sensitive. Invasive bladder cancer patients expressed significantly more CDK6 cytoplasmically and unclearly than adjacent tissues^26^. Our present study shows a similar result: basal bladder cancers express higher levels of CDK6. Additionally, cyclin pathway gene alterations accompanied by FGF/FGFR aberrations are very common in urinary tract tumors^27^. Blocking the cyclin pathway gene and other relevant molecular pathways may therefore provide a potential treatment option for patients suffering from basal bladder cancer.

There has been rapid progress in immune checkpoint molecule inhibitor development in recent years. These inhibitors have provided a promising opportunity for immunotherapy of bladder cancer, particularly for muscle-invasive and metastatic bladder cancers^28^, ^29^. However, only a limited number of patients have a favorable response to immune checkpoint inhibitors, which might be due to incomplete infiltration of the immunotherapeutic agent and immune suppression by tumor microenvironment^30^. According to growing evidence, tumor immune microenvironments play a significant role in surveillance and immunity^31^.

It is reported that combined treatment with CDK4/6 inhibitor and anti-PD-1 antibody could improve synergistic anti-tumor effect via promoting immune infiltration in a B cell-dependent manner^32^. Consistently, CDK4/6 inhibitor enhances anti-tumor effect of chemotherapy and immune checkpoint inhibitor combinations via enhancing T-cell activation^33^. Signal transduction of T cell is activated to enhance T cell-mediated immunotherapy by CDK6 depletion, which reshapes the tumor immune microenvironment^34^. Moreover, CDK6 inhibition has been shown to increase the infiltration and activity of cytotoxic T lymphocytes (CTLs) within the tumor microenvironment, thereby amplifying the anti-tumor immune response. This effect is thought to be mediated through the upregulation of major histocompatibility complex (MHC) class I molecules and the downregulation of immune suppressive cytokines, creating a more favorable environment for T cell-mediated tumor destruction^35^, ^36^. Further studies show that inhibition of CDK6 can lead to a decreased expression of PD-L1 and other immune checkpoints, potentially sensitizing tumors to immune checkpoint blockade therapies^37^. This suggests that combining CDK6 inhibitors with immune checkpoint inhibitors may have a synergistic effect, improving the overall efficacy of immunotherapy^38^. In conclusion, targeting CDK6 in combination with immunotherapy represents a promising strategy for enhancing anti-tumor immune responses and overcoming resistance to current treatments^39^, ^40^.

According to the results in our study, the elevated expression of CDK6 has been observed to correlate with increased immune infiltration in patients, accompanied by a proliferation of immune checkpoints. This suggests that despite the significant immune infiltration characteristics in this patient cohort, there is a manifestation of an immune exhaustion phenotype. Such immune exhaustion might be a critical factor contributing to treatment resistance. Intriguingly, while immunotherapy is generally more effective in patients with rich immune infiltration, the response to immunotherapy in patients with high CDK6 expression is comparatively poor. Further analysis revealed that in BLCA, CDK6 is significantly correlated not only with various immune regulatory genes and immune checkpoint genes but also with the infiltration of different types of immune cells. Specifically, in BLCA, CDK6 expression shows a significant positive correlation with 60 immune checkpoint genes, indicating that CDK6 may play a critical role in the immune microenvironment of bladder cancer. Moreover, the high CDK6 expression group activated most steps in the immunity cycle, except for cancer antigen presentation (Step 2) and the trafficking of immune cells to tumors (Step 4), suggesting that CDK6 has a broad impact on regulating immune responses. This immune exhaustion phenotype may explain why patients with high CDK6 expression have a poor response to immunotherapy. Although these patients exhibit high immune infiltration, the immune cells may be functionally exhausted and unable to effectively attack tumor cells. This provides new insights for further research on CDK6 as a target for immunotherapy. Inhibiting CDK6 function may help restore immune cell activity and improve the efficacy of immunotherapy. Additionally, the significant positive correlation between high CDK6 expression and immune checkpoint genes in bladder cancer suggests that a combined treatment strategy using CDK6 inhibitors and immune checkpoint inhibitors may enhance the response rate to immunotherapy. This combined treatment approach may offer a more effective therapy by reducing immune exhaustion and enhancing the anti-tumor activity of immune cells. In conclusion, CDK6 plays a crucial role in immune regulation, particularly in bladder cancer. Research and therapeutic strategies targeting CDK6 not only have the potential to reveal mechanisms of tumor immune evasion but also may provide new targets and methods for improving the efficacy of immunotherapy.

Nevertheless, there were still some limitations to our study. Firstly, the samples of patients from one clinical center might lead to some sample bias. Secondly, our study was retrospective, which introduces limitations such as potential confounding factors and constraints in establishing causality, and further validation of our conclusions necessitates the design of both *in vivo* and *in vitro* experiments. As a conclusion, it is necessary to conduct follow-up studies to ascertain the precise function and mechanism of action of CDK6.

## 5. Conclusion

In summary, our study has highlighted the critical role of CDK6 in the progression and prognosis of bladder cancer. CDK6 is significantly associated with poor clinical outcomes, advanced pathological stages, and an immune-exhausted tumor microenvironment in bladder cancer patients. There is evidence to suggest that CDK6 could serve as a prognostic biomarker and a therapeutic target. Given the limited efficacy of current immunotherapies for a substantial proportion of bladder cancer patients, integrating CDK6 inhibitors with immune checkpoint inhibitors may offer a promising approach to overcome resistance and enhance anti-tumor immune responses.

## Supplementary Material

Supplementary figures and tables.

## Abbreviations

CDK6: Cyclin-dependent kinase 6
BLCA: Bladder cancer
TCGA: The Cancer Genome Atlas
DEGs: Differentially Expressed Genes
GO: Gene Ontology
GSEA: Gene Set Enrichment Analysis
BP: Biological Process
CC: Cellular Component
MF: Molecular Function
IC50: half maximal inhibitory concentration
KEGG: Kyoto Encyclopedia of Genes and Genomes
GBML: Glioblastoma Multiforme
LGG: Low-Grade Glioma
PAAD: Pancreatic Adenocarcinoma
MESO: Mesothelioma
ACC: Adrenocortical Carcinoma
OS: overall survival
RFS: recurrence-free survival
TIME: tumor immune microenvironment
GZMH: Granzyme H
GZMA: Granzyme A
GZMB: Granzyme B
GZMM: Granzyme M
PRF1: Perforin 1
IFNG: Interferon Gamma
IDH1: Isocitrate Dehydrogenase 1
BRFA: B-Raf Proto-Oncogene
KDM6A: Lysine K Demethylase 6A
SYNE1: Spectrin Repeat Containing Nuclear Envelope Protein 1
CREBBP: CREB Binding Protein
DST: Dystonin
MYCN: N-Myc Proto-Oncogene
FGF: Fibroblast Growth Factor
FGER: Fibroblast Growth Factor Receptor
