# Supplementary material for: CDK6 as a Biomarker for Immunotherapy, Drug Sensitivity, and Prognosis in Bladder Cancer: Bioinformatics and Immunohistochemical Analysis
Source: Int J Med Sci. 2024 Sep 16;21(12):2414–29. doi: 10.7150/ijms.101043 (PMC11413897; doi:10.7150/ijms.101043)
Supplement: Supplementary file 1 — Supplementary figures and tables. [file ijmsv21p2414s1.pdf]

Supplementary figure S1

A

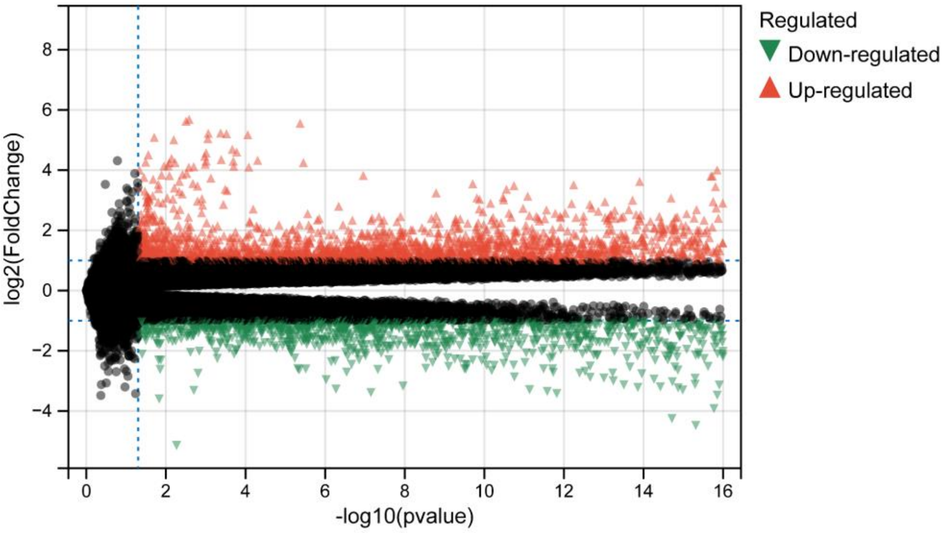

B

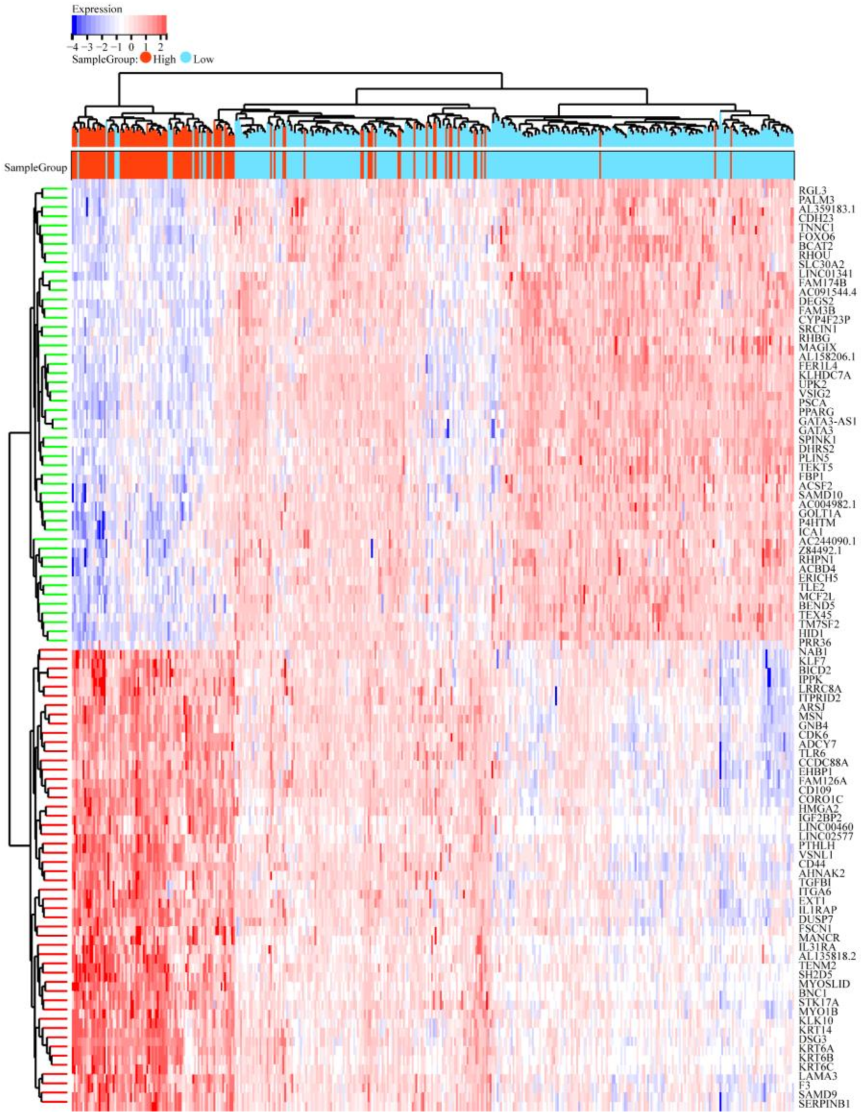

Supplementary figure S2

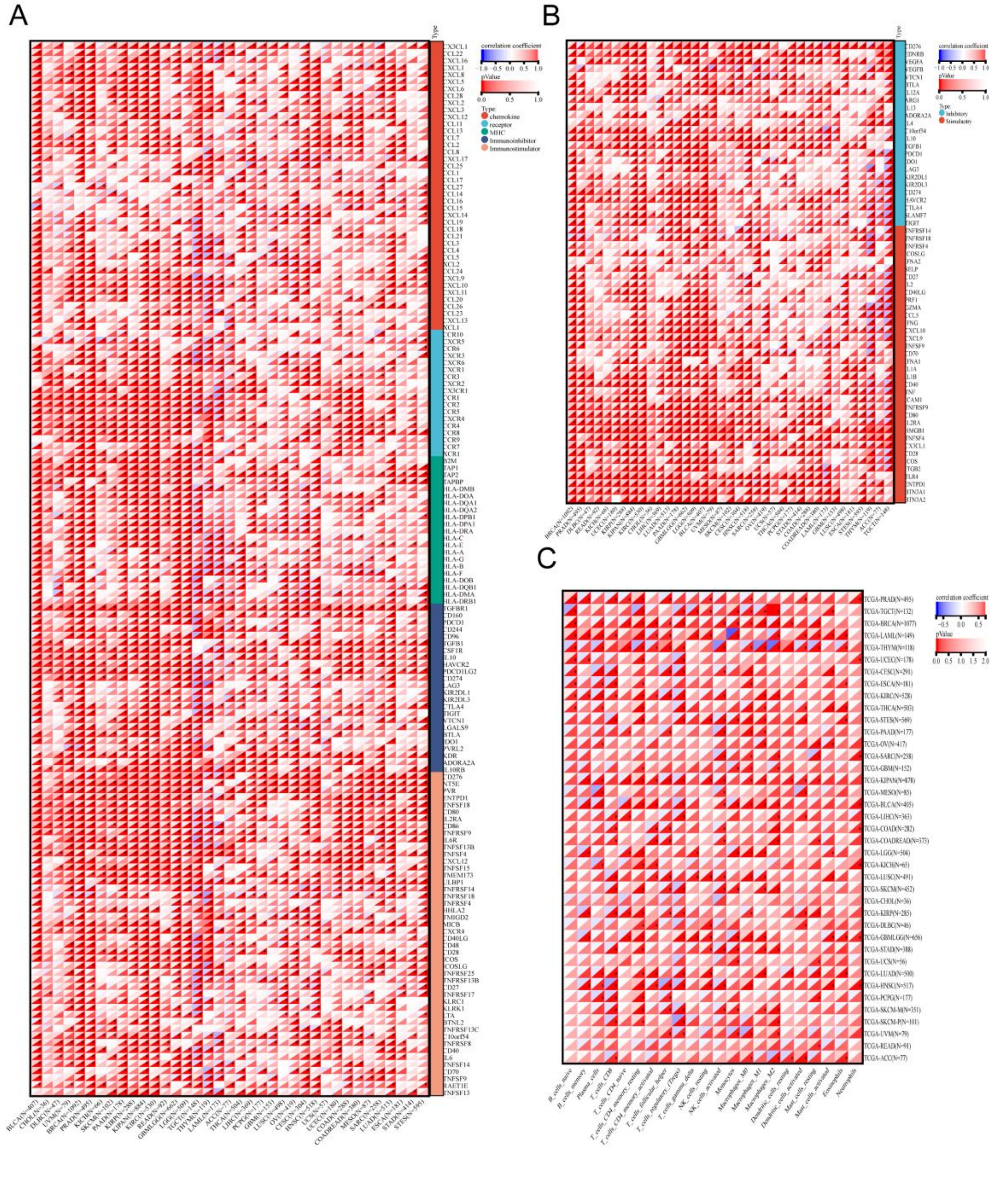

Table S1

TABLE S1 | Clinical information of patients in cohort 1. Bold indicates p-values less than 0.05.

Characteristics	Low(N=302)	High(N=106)	Total(N=408)	p-value
Age				0.60
<60	62(15.20%)	25(6.13%)	87(21.32%)	
>=60	240(58.82%)	81(19.85%)	321(78.68%)	
Sex				0.09
Female	72(17.65%)	35(8.58%)	107(26.23%)	
Male	230(56.37%)	71(17.40%)	301(73.77%)	
Grade				**0.01**
High	279(68.89%)	105(25.93%)	384(94.81%)	
Low	21(5.19%)	0(0.0e+0%)	21(5.19%)	
Unable to evaluate			3	
Pathologic stage				**0.04**
I	2(0.49%)	0(0.0e+0%)	2(0.49%)	
II	103(25.37%)	27(6.65%)	130(32.02%)	
III	92(22.66%)	48(11.82%)	140(34.48%)	
IV	104(25.62%)	30(7.39%)	134(33.00%)	
Unable to evaluate			2	

Table S2

TABLE S2 | Clinical information of patients in cohort 2. Bold indicates p-values less than 0.05.

Characteristics	Low(N=50)	High(N=50)	Total(N=100)	p-value
Age				0.21
<60	14(14.00%)	21(21.00%)	35(35.00%)	
>=60	36(26.00%)	29(29.00%)	65(65.00%)	
Sex				0.55
Female	8(8.00%)	5(5.00%)	13(13.00%)	
Male	42(42.00%)	45(45.00%)	87(87.00%)	
Pathologic stage				0.55
I/II	45(45.00%)	42(42.00%)	87(87.00%)	
III/IV	5(5.00%)	8(8.00%)	13(13.00%)	

Table S3

TABLE S3 | Clinical information of patients in cohort 3. Bold indicates p-values less than 0.05.

Characteristics	Low(N=191)	High(N=157)	Total(N=348)	p-value
Sex				0.27
Female	37(10.63%)	39(11.21%)	76(21.84%)	
Male	154(44.25%)	118(33.91%)	272(78.16%)	
Binary Response				**0.02**
CR/PR	46(15.44%)	22(7.38%)	68(22.82%)	
SD/PD	115(38.59%)	115(38.59%)	230(77.18%)	
Others			50	
Enrollment IC				**0.02**
IC0	63(18.10%)	36(10.34%)	99(28.45%)	
IC1	75(21.55%)	57(16.38%)	132(37.93%)	
IC2	53(15.23%)	64(18.39%)	117(33.62%)	
Immune phenotype				**7.3e-3**
Desert	48(16.90%)	28(9.86%)	76(26.76%)	
Excluded	81(28.52%)	53(18.66%)	134(47.18%)	
Inflamed	30(10.56%)	44(15.49%)	74(26.06%)	
Others			64	

Table S4

Characteristics	Low(N=302)	High(N=106)	Total(N=408)	p-value
Baylor subtype				1.7e-31
Basal	56(13.73%)	87(21.32%)	143(35.05%)	
Differentiated	246(60.29%)	19(4.66%)	265(64.95%)	
UNC subtype				2.3e-26
Basal	86(21.08%)	94(23.04%)	180(44.12%)	
Luminal	216(52.94%)	12(2.94%)	228(55.88%)	
CIT subtype				1.9e-27
MC1	154(37.75%)	8(1.96%)	162(39.71%)	
MC2	24(5.88%)	1(0.25%)	25(6.13%)	
MC3	17(4.17%)	1(0.25%)	18(4.41%)	
MC4	36(8.82%)	3(0.74%)	39(9.56%)	
MC5	1(0.25%)	0(0.0e+0%)	1(0.25%)	
MC6	3(0.74%)	1(0.25%)	4(0.98%)	
MC7	67(16.42%)	92(22.55%)	159(38.97%)	
Lund subtype				2.1e-30
Ba/Sq	11(2.70%)	42(10.29%)	53(12.99%)	
Ba/Sq-Inf	20(4.90%)	24(5.88%)	44(10.78%)	
GU	31(7.60%)	1(0.25%)	32(7.84%)	
GU-Inf	26(6.37%)	3(0.74%)	29(7.11%)	
Mes-like	25(6.13%)	9(2.21%)	34(8.33%)	
Sc/NE-like	11(2.70%)	6(1.47%)	17(4.17%)	
Uro-Inf	22(5.39%)	1(0.25%)	23(5.64%)	
UroA-Prog	87(21.32%)	7(1.72%)	94(23.04%)	
UroB	11(2.70%)	13(3.19%)	24(5.88%)	
UroC	58(14.22%)	0(0.0e+0%)	58(14.22%)	
MDA subtype				7.6e-28
basal	59(14.46%)	84(20.59%)	143(35.05%)	
luminal	138(33.82%)	7(1.72%)	145(35.54%)	
p53-like	105(25.74%)	15(3.68%)	120(29.41%)	
TCGA subtype				5.7e-29
Basal squamous	51(12.50%)	82(20.10%)	133(32.60%)	
Luminal	45(11.03%)	1(0.25%)	46(11.27%)	
Luminal infiltrated	62(15.20%)	5(1.23%)	67(16.42%)	
Luminal papillary	134(32.84%)	12(2.94%)	146(35.78%)	
Neuronal	10(2.45%)	6(1.47%)	16(3.92%)	

Table S5

Characteristics	High(N=157)	Low(N=191)	Total(N=348)	p-value
Baylor subtype				2.7e-16
Basal	132(37.93%)	77(22.13%)	209(60.06%)	
Differentiated	25(7.18%)	114(32.76%)	139(39.94%)	
UNC subtype				1.6e-16
Basal	108(31.03%)	46(13.22%)	154(44.25%)	
Luminal	49(14.08%)	145(41.67%)	194(55.75%)	
CIT subtype				3.2e-12
MC1	20(5.75%)	79(22.70%)	99(28.45%)	
MC2	4(1.15%)	6(1.72%)	10(2.87%)	
MC3	2(0.57%)	15(4.31%)	17(4.89%)	
MC4	53(15.23%)	48(13.79%)	101(29.02%)	
MC5	2(0.57%)	8(2.30%)	10(2.87%)	
MC6	0(0.0e+0%)	2(0.57%)	2(0.57%)	
MC7	76(21.84%)	33(9.48%)	109(31.32%)	
Lund subtype				5.6e-17
Ba/Sq	31(8.91%)	8(2.30%)	39(11.21%)	
Ba/Sq-Inf	22(6.32%)	8(2.30%)	30(8.62%)	
GU	1(0.29%)	26(7.47%)	27(7.76%)	
GU-Inf	8(2.30%)	23(6.61%)	31(8.91%)	
Mes-like	31(8.91%)	18(5.17%)	49(14.08%)	
Sc/NE-like	6(1.72%)	5(1.44%)	11(3.16%)	
Uro-Inf	14(4.02%)	16(4.60%)	30(8.62%)	
UroA-Prog	15(4.31%)	51(14.66%)	66(18.97%)	
UroB	22(6.32%)	5(1.44%)	27(7.76%)	
UroC	7(2.01%)	31(8.91%)	38(10.92%)	
MDA subtype				1.3e-19
basal	82(23.56%)	19(5.46%)	101(29.02%)	
luminal	24(6.90%)	100(28.74%)	124(35.63%)	
p53-like	51(14.66%)	72(20.69%)	123(35.34%)	
TCGA subtype				4.4e-16
Basal squamous	81(23.28%)	21(6.03%)	102(29.31%)	
Luminal	5(1.44%)	22(6.32%)	27(7.76%)	
Luminal infiltrated	52(14.94%)	91(26.15%)	143(41.09%)	
Luminal papillary	14(4.02%)	54(15.52%)	68(19.54%)	
Neuronal	5(1.44%)	3(0.86%)	8(2.30%)	
